# Transcript profiling reveals expression differences in wild-type and glabrous soybean lines

**DOI:** 10.1186/1471-2229-11-145

**Published:** 2011-10-26

**Authors:** Matt Hunt, Navneet Kaur, Martina Stromvik, Lila Vodkin

**Affiliations:** 1 Department of Crop Sciences, University of Illinois, Urbana, Illinois, 61801, USA; 2 Department of Plant Science/McGill Centre for Bioinformatics, McGill University, Macdonald campus, Ste-Anne-de-Bellevue, QC H9X 3V9, Canada; 3 Current address: Ohio State University, Columbus, OH 43210, USA

## Abstract

**Background:**

Trichome hairs affect diverse agronomic characters such as seed weight and yield, prevent insect damage and reduce loss of water but their molecular control has not been extensively studied in soybean. Several detailed models for trichome development have been proposed for *Arabidopsis thaliana*, but their applicability to important crops such as cotton and soybean is not fully known.

**Results:**

Two high throughput transcript sequencing methods, Digital Gene Expression (DGE) Tag Profiling and RNA-Seq, were used to compare the transcriptional profiles in wild-type (cv. Clark standard, CS) and a mutant (cv. Clark glabrous, i.e., trichomeless or hairless, CG) soybean isoline that carries the dominant *P1 *allele. DGE data and RNA-Seq data were mapped to the cDNAs (Glyma models) predicted from the reference soybean genome, Williams 82. Extending the model length by 250 bp at both ends resulted in significantly more matches of authentic DGE tags indicating that many of the predicted gene models are prematurely truncated at the 5' and 3' UTRs. The genome-wide comparative study of the transcript profiles of the wild-type versus mutant line revealed a number of differentially expressed genes. One highly-expressed gene, *Glyma04g35130*, in wild-type soybean was of interest as it has high homology to the cotton gene *GhRDL1 *gene that has been identified as being involved in cotton fiber initiation and is a member of the BURP protein family. Sequence comparison of *Glyma04g35130 *among Williams 82 with our sequences derived from CS and CG isolines revealed various SNPs and indels including addition of one nucleotide C in the CG and insertion of ~60 bp in the third exon of CS that causes a frameshift mutation and premature truncation of peptides in both lines as compared to Williams 82.

**Conclusion:**

Although not a candidate for the *P1 *locus, a BURP family member (*Glyma04g35130*) from soybean has been shown to be abundantly expressed in the CS line and very weakly expressed in the glabrous CG line. RNA-Seq and DGE data are compared and provide experimental data on the expression of predicted soybean gene models as well as an overview of the genes expressed in young shoot tips of two closely related isolines.

## Background

Plant trichomes are appendages that originate from epidermal cells and are present on the surface of various plant organs such as leaves, stems, pods, seed coats, flowers, and fruits. Trichome morphology, varying greatly among species, includes types that are unicellular, multicellular, glandular, non-glandular (as in soybean), single stalks (soybean), or branched structures (Arabidopsis) [1]. Various functions have been ascribed to trichomes, including roles as attractants of pollinators, in protection from herbivores and UV light, and in transpiration and leaf temperature regulation [2-4].

The genetic control of non-glandular trichome initiation and development has been studied extensively in Arabidopsis and cotton. In Arabidopsis, several genes were identified that regulate trichome initiation and development. A knockout of *GLABRA1 *(*GL1*) results in glabrous Arabidopsis plants [5]. The *GL1 *encodes a R2R3 MYB transcription factor that binds either GL3 or ENHANCER OF GLABRA3 (EGL3), basic helix-loop-helix (bHLH) transcription factors, which in turn bind to TRANSPARENT TESTA GLABRA (TTG) protein, a WD40 transcription factor [6,7]. The binding of GL1-GL3/EGL3-TTG1 forms a ternary complex, which initiates the progression of an epidermal cell development into a trichome by binding to the *GLABRA2 *(*GL2*) gene, which encodes a homodomain/leucine zipper transcription factor [8].

Microarray gene expression analysis of two Arabidopsis mutants lacking trichomes with wild-type Arabidopsis trichomes identified several cell-wall related up-regulated genes [9]. Transcriptome analyses of wild-type trichomes and the double mutant *gl3-sst sim *trichomes in Arabidopsis identified four new genes: *HDG2*, *BLT*, *PEL3*, and *SVB *that are potentially associated with trichome development [10].

Cotton fibers are single celled trichomes that develop from the surface of cotton seed [11]. The development of cotton fibers goes through four stages of development: differentiation/fiber initiation, expansion/elongation, secondary cell wall biosynthesis, and maturity [11,12]. Unlike Arabidopsis, the specific genes/proteins involved in cotton fiber initiation have not been clearly elucidated. Several different approaches have been taken to study cotton fiber initiation and elongation, including studying gene expression in normal fibers [12-14], comparing gene expression in fiber development mutants to normal cotton varieties [13,15-17], and using existing EST or gene sequences from cotton or Arabidopsis clones [18-23].

Microarray studies comparing cotton fiber initiation mutants identified six clones falling into either BURP-containing protein or RD22-like protein that were over expressed in cotton fibers in wild-type compared with the mutant lines [15,16]. These six clones are all members of the BURP domain gene family as the RD22 protein that was identified in Arabidopsis is also a member of the BURP domain family of proteins [24].

Soybean has 23 possible BURP domain containing genes which are classified into five subfamilies: BNM2-like, USP-like, RD22-like, PG1β-like, and BURPV (a new subfamily) depending on the translated products homology to these founding members of the BURP family [25,26]. BURP genes are plant-specific and with diverse functions in plants [24,25].

Unlike Arabidopsis and cotton, the developmental genetics of soybean trichomes has not been studied extensively. However, there are several soybean trichome developmental mutants available, including *P1 *(glabrous), *pc *(curly pubescence), *Pd *(dense pubescence), *Ps *(sparse pubescence), and *p2 *(puberulent) that are each controlled by a different single Mendelian locus [27]. These mutants have been used to relate the importance of trichome to insect resistance [4,28,29], evapotranspiration [2,30,31] and other yield related characteristics. However, until now, none of these glabrous classical mutations has been studied at the molecular level. We studied the dominant *P1 *glabrous soybean mutant using two high throughput transcript sequencing technologies to reveal major expression differences between the two genotypes. RNA and DNA blots further characterized a highly differentially expressed BURP family member *Glyma04g35130 *that varied between the two genotypes and may be associated with trichome development in soybean although it is not a candidate for the *P1 *locus.

## Results

### DGE library construction and identification of authentic tags

We first used Illumina DGE Tag Profiling to determine the differential gene expression between wild-type Clark standard (CS) and glabrous-mutant Clark glabrous (CG) in shoot tip tissue. The CG isoline was developed by backcrossing the *P1 *glabrous mutant into Clark for six generations [27]. Total RNA isolated from shoot tips of both CS and CG plants was analyzed by Illumina DGE tag profiling to create transcriptome profiles of the two isolines. DGE tags are 16-nucleotide long and are designed to be derived from the 3'UTR of the transcript. DGE data provide a quantitative measure of transcript abundance in the RNA population and can also identify previously unannotated genes. The majority of DGE tags are expected to match only one location in the genome, with the remaining tags matching duplicated genes, alternate transcripts, antisense strands, or repeated sequences [32].

**Figure 1:**
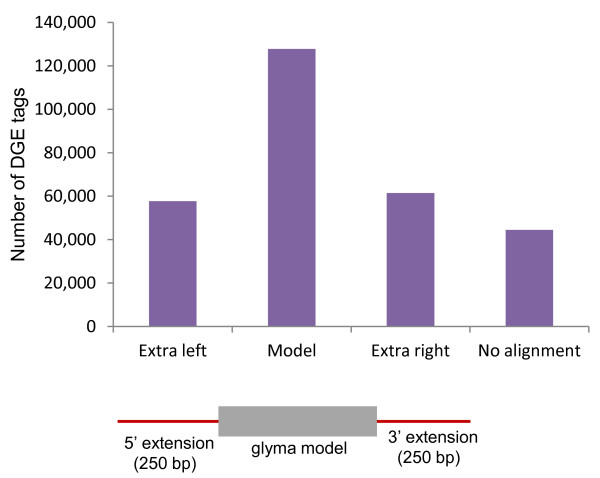
**Distribution of DGE 16-bp tags according to their positional alignment to the Williams 82 Glycine max gene models**. The cDNA models were downloaded from Phytozome [34]. Shown are the number of tags that matched to either the cDNA model or to 250 bases extended to the 5' or 3' end of each model as represented by the figure underneath the graph.

We obtained a total of 5.28 and 5.26 million tags from the CS and CG lines respectively, that resulted in approximately 84,899 and 85,402 unique tags from the CS and CG lines, which had counts of 5 tags or more in at least one library. DGE tags were aligned to the 78,774 cDNA gene models (known as Glyma models) predicted from the soybean reference genome of cv. Williams 82 [33] and available from Phytozome v.6 [34] using Bowtie [35]. With a stringent criterion of 0 mismatches within the 16-nucleotide tag alignments, most of the tags aligned to the models but large numbers of tags did not. In order to retrieve alignments in the cases where the computationally predicted Glyma models did not call sufficient 3'UTR sequence, we extended the Glyma models at both the 5' and 3' ends by 250 bases in each direction. This analysis produced more hits of tags that corresponded to the extra left, junction left, junction right, and extra right region in addition to the model (Figure 1 & Additional file 1). These data show that the current computational models from the soybean genome are likely incomplete for especially for the 3' end. Of the approximately 5.2 million tags in each library, we found that 4.7 million aligned to one or more of the extended soybean genome models. The remainder showed no alignment to any model or to the extended Glyma models. Non-aligned sequences might be attributed at least partially to single nucleotide differences in the soybean cultivars used in this study (Clark) as compared to the references soybean genome (cv. Williams 82) since a 0 mismatch criteria was used in the alignments.

**Figure 2:**
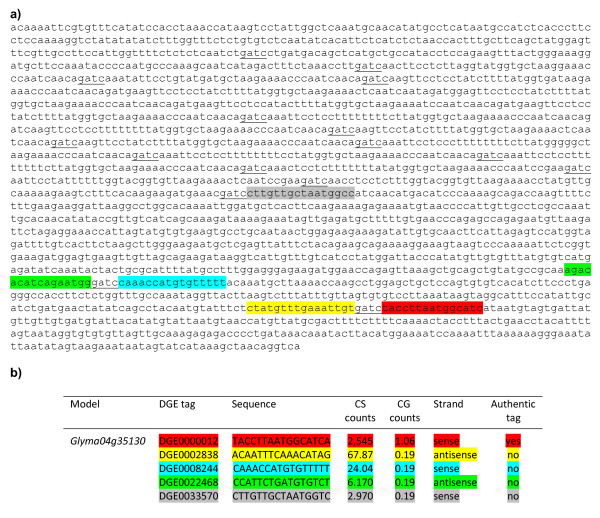
**Identification of the authentic tag corresponding to its Glyma model**. (A) Clark standard (CS) *Glyma04g35130 *transcript sequence. *Underlined *sequences represent *Dpn*II restriction sites. DGE0000012, indicated in *red *is an authentic tag because it is adjacent to the last *Dpn*II site in the 3'UTR sequence of this gene. Other non-authentic site tags on either the sense or antisense strand are also shown: DGE0002838 (*yellow*) and DGE0022468 (*green*) originated from restriction fragments which are not washed after digestion of cDNA with *Dpn*II; DGE0008244 (*ferozi*) and DGE0033570 (*grey*) originated due to inefficient restriction of cDNA by *Dpn*II. (B) Five DGE tags match *Glyma04g35130 *sequence. Their respective sequences and counts in CS and the glabrous-mutant (CG) are indicated.

An example that illustrates multiple DGE tags found in a single Glyma model is *Glyma04g35130*, that matches five DGE tags: DGE0000012, DGE0002838, DGE0008244, DGE0022468, and DGE0033570 (Figure 2A &2B). Out of these 5 tags, only DGE0000012 originates from the authentic position within *Glyma04g35130 *because this tag sequence is adjacent to the last *Dpn*II site in 3'UTR and additionally its abundance represents a normalized count of 2545 tags per million aligned DGE reads in the CS line as compared to other less abundant tags that likely originate from incomplete restriction digestion of *Dpn*II sites on either the positive or negative strands. For example, DGE0002838 and DGE0022468 likely originate from restricted fragments, which were not washed away after digestion of cDNA with *Dpn*II (Figure 2). DGE0008244 and DGE0033570 originate due to inefficient restriction by *Dpn*II (Figure 2). Thus, DGE0000012 is the authentic tag representing the transcript for *Glyma04g35130 *(Figure 2A &2B). As will be discussed later, the abundance of transcripts originating from the authentic DGE tag position DGE0000012 is very high in CS and dramatically reduced in CG (CS/CG = 2,545/1.06 tags). Additionally, all of the less abundant secondary tags from different positions showed much lower counts in the CG line, indicating that they all arise from the same Glyma model, *Glyma04g35130*. One DGE tag can also match to more than one Glyma model. For instance, DGE0004659 matches two Glyma models: *Glyma03g41750 *and *Glyma19g44380 *(data not shown). This DGE0004659 tag originates from *Glyma19g44380 *because the sequence of this DGE tag is adjacent to the last *Dpn*II site in its 3'UTR as expected according to the protocol used for mRNA sequencing by Illumina.

### Transcriptome comparison of Clark standard and Clark glabrous with DGE tag profiling

**Figure 3:**
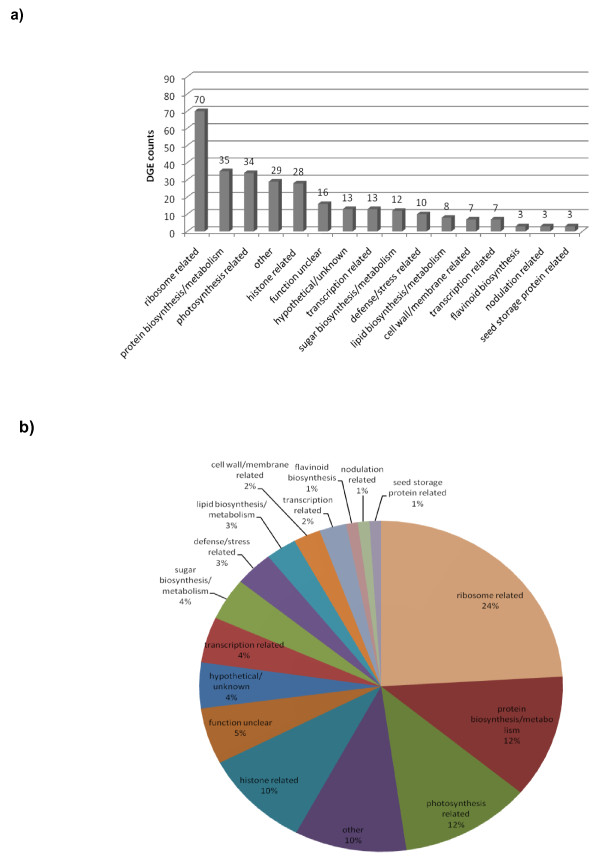
**Distribution of the top 300 highly-expressed DGE tags among their functional categories.** (A) The top 300 most abundant DGE tags in Clark standard (CS) and Clark glabrous (CG) separated into functional categories. (B) Percentage distribution of the functional categories of the genes corresponding to the top 300 most abundant DGE tags in both Clark standard (CS) and Clark glabrous (CG).

Approximately 85,000 unique tags representing over 4.7 million DGE tags that aligned to the extended Glyma cDNA predicted gene models of the soybean genome were generated from each line of the CS and CG isolines and counts were normalized per million aligned (mapped) reads. The resulting transcriptome datasets identified highly expressed genes as well as differentially expressed genes between young shoot tips of CS and CG isolines. The top 300 highly expressed genes (Additional file 2) in both genotypes were divided into 15 broad functional categories (Figure 3A) and their percentage distribution is illustrated in Figure 3B. As shown in Figure 3A, the genes from the top 5 categories that were highly expressed in shoot tip of CS and CG encode proteins related to: ribosomes (70 different tags), protein biosynthesis/metabolism (35 tags), photosynthesis (34 tags), other (29 tags), and histones (28 tags). In addition to automated annotations to the soybean references genome [34] and other databases, the annotation of these DGE tags were verified manually using blast searches to the soybean EST databases as described in the Materials and Methods section. The matches to specific ESTs are shown in the Additional File 2. This approach also verified direct expression of the DGE tags that were located in the extended Glyma model regions.

Tags that were either ≥2-fold over or under-expressed in CS in comparison with CG with a minimum of 42 counts per tag per million mapped reads were also analyzed in greater detail. Of these, 144 (Additional file 3) showed ≥2-fold over-expression in CS as compared to CG and 23 were under-expressed in CS. Of those, some showing the greatest differential expression (either over or under-expressed relative to the Clark standard line) are shown in Table 1.

**Table 1:** Top DGE tags and RNA-Seq RPKM for genes that are over expressed either in Clark standard (a) or Clark glabrous (b).

**a)**					**DGE**			**RNASeq**	
				
	**DGE Tag ID **	**Glyma Model **	**Annotation **	**CS**	**CG**	**CS/CG**	**CS**	**CG**	**CS/CG**
	
	DGE0000165	Glyma14g04140.1	copper ion binding protein	595.96	0.21	2801	4.58	2.31	1.98
	DGE0000012	Glyma04g35130.1	BURP domain protein	2544.7	1.06	2392	480.38	0.01	45679.50
	DGE0000974	Glyma16g02940.1	chitinase	164.04	0.21	771	139.37	91.88	1.52
	DGE0002509	no Glyma model	cyclic nucleotide-gated channel B	75.53	0.19	394.44	NA	NA	NA
	DGE0003828	no Glyma model	small polyprotein 2	51.49	0.19	268.89	NA	NA	NA
	DGE0003923	Glyma16g28030.1*	chlorophyll a-b binding protein 1	50.43	0.19	263.33	1093.27	280.90	3.89
	DGE0001116	Glyma08g22680.1	Blue copper protein precursor	146.17	1.06	137.4	4.39	0.44	10.02
	DGE0002248	Glyma11g07850.1	cytochrome P450 monooxygenase CYP84A16	82.77	4.04	20.474	7.29	0.34	21.44
	DGE0002191	Glyma15g15660.1	putative allergen	84.26	4.26	19.8	5.55	1.38	4.03
**b)**									
	DGE0002073	Glyma09g38410.1	calreticulin-3 precursor	88.94	329.79	0.2697	10.35	21.32	0.49
	DGE0000639	Glyma07g05620	phosphatidylserine decarboxylase invertase/pectin methylesterase inhibitor family	233.83	753.40	0.3104	3.07	65.57	0.05
	DGE0004450	Glyma06g47740.1	protein	44.89	143.62	0.31	8.04	28.56	0.28
	DGE0000888	Glyma05g09160.1	lipid transfer protein	177.87	567.45	0.31	7.03	12.47	0.56
	DGE0003408	Glyma02g01250.1	hypothetical protein invertase/pectin methylesterase inhibitor family	57.021	177.66	0.32	3.67	4.13	0.89
	DGE0002491	Glyma06g47740.1	protein	75.74	233.40	0.32	8.04	28.56	0.28
	DGE0002716	Glyma13g09420.1	putative wall-associated kinase	70.64	185.53	0.38	10.29	13.44	0.77
	DGE0002161	Glyma03g32820.1	glycine-rich protein	85.11	207.45	0.41	1.21	3.85	0.31
	DGE0001547	Glyma05g02630.1	zinc ion binding protein	114.47	264.89	0.43	8.19	12.54	0.65
	DGE0002544	Glyma01g07860.1	copper amine oxidase	74.47	167.23	0.45	37.11	251.18	0.15
	DGE0002615	Glyma06g17860.1	putative diphosphonucleotide phosphatase	72.98	158.72	0.46	33.91	224.33	0.15
	DGE0003965	Glyma02g37610.1	Aspartic proteinase nepenthesin-1 precursor	50	108.30	0.46	0.55	1.90	0.29
	DGE0002836	no Glyma model	root nodule extensin	67.87	137.66	0.49	NA	NA	NA
	DGE0004693	Glyma10g35870.1	auxin down-regulated protein	42.55	85.74	0.50	40.61	209.40	0.19
	DGE0001864	Glyma12g36160.1	receptor-like protein kinase	97.45	196.17	0.50	23.48	27.61	0.85

DGE is normalized per million tags and RNA-Seq is shown in RPKM *glyma model has SNP in their tag sequence.

Among the tags overexpressed in the CS line, one particular tag corresponds to a gene located on *Glyma04 *chromosome, specifically *Glyma04g35130*, and showed >2000-fold expression difference between CS/CG = 2,545/1.06 tags per million aligned tags (Table 1). The *Glyma04g35130 *gene is a member of the *BURP *gene family. It has high homology to the cotton gene- *RESISTANCE TO DROUGHT RD22-like 1 *(*GhRDL1*), involved in cotton fiber initiation and member of the BURP protein domain family [15,16]. Soybean has a total of 23 BURP domain containing genes and BURP gene family members from other species are known to have diverse functions [26]. Some of the proposed functions of BURP family members include: regulation of fruit ripening in tomato [36,37], response to drought stress induced by abscisic acid in Arabidopsis [38], tapetum development in rice [39], and seed coat development in soybean [40]. In Clark, the DGE0000012 tag found to correspond to *Glyma04g35130 *is the 12^th ^most abundant tag in the DGE data set. For perspective, the 4^th ^most abundant tag with a normalized count of 4,903 tags matches a chlorophyll a/b binding factor as do several of the most abundant tags (Additional file 2).

For further verification of differential expression, we used DESeq package in R without replications as described [41]. This condition relies on the assumption that in the isolines most genes will be similarly expressed, thus treating the two lines as repeats. This analysis produced the same list of significant up and down-regulated genes. Lists of all differentially expressed genes in CS versus CG or vice versa are shown in Additional file 4A &4B, respectively, using the DESeq package.

### Comparison of DGE data with RNA-Seq

The sequencing of CS and CG transcriptome by RNA-Seq generated 91.4 and 88.7 million 75-bp reads, respectively from an independent biological sample of the CS and CG shoot tips. These tags were mapped to the 78,744 soybean gene models using Bowtie [35]. RNA-Seq data was normalized in reads per kilo base of gene model per million mapped reads (RPKM) as the sensitivity of RNA-Seq depends on the transcript length [42]. RNA-Seq analysis revealed that at the cutoff point of 10 RPKM, a total of 11,574 and 14,378 genes were expressed in CS and CG, respectively. At a cutoff of 1 RPKM, however, 41,972 and 44,120 genes were expressed in CS and CG, respectively. Together, the results suggest that in the RNA-Seq transcriptome, ~50% of genes are expressed in both wild-type and mutant soybean.

The genes that showed over expression in CS compared to CG or vice versa in DGE data were compared with RNA-Seq data. Table 1 shows some of the RNA-Seq data compared to the DGE data that have the same trend, i.e. over or under expression in CS relative to CG. Among the BURP genes, RNA-Seq data has enabled nearly the same trend of differential expression and has confirmed that *Glyma04g35130 *BURP gene is over expressed in CS compared to CG. Similarly, among the seven BURP genes, four genes: *Glyma04g35130, Glyma07g28940, Glyma14g20440*, and *Glyma14g20450 *showed a same trend in both RNA-Seq and DGE data (Table 2).

**Table 2:** Expression of BURP gene family members as measured by DGE and RNA-Seq.

			DGE			RNASeq		
				
			Norm Counts		Ratio	RPKM		Ratio
				
BURP genes	e-value	DGE tags	CS	CG	CS/CG	CS	CG	CS/CG
Glyma04g35130	0	DGE0000012	2544.68	1.06	2392.00	480.38	0.01	45679.50
Glyma07g28940	4.4E-43	no tag	0.00	0.00	0.00	2.86	1.07	2.68
Glyma04g08410	1.4E-30	DGE0060859	0.85	11.70	0.07	1.43	0.48	2.99
Glyma14g20450	7.5E-15	DGE0001112	147.02	80.64	1.82	0.00	0.00	0.00
Glyma06g08540	3.2E-13	DGE0060859	0.85	11.70	0.07	66.07	6.79	9.73
Glyma14g20440	3.2E-13	DGE0002418	78.09	24.68	3.16	51.77	10.97	4.72
Glyma06g01570	3.60E-06	DGE0000631	236.38	248.51	0.95	0.56	0.26	2.14

### RNA blots confirm the dramatic transcript level differences of *Glyma04 BURP *gene in Clark standard and Clark glabrous

**Figure 4:**
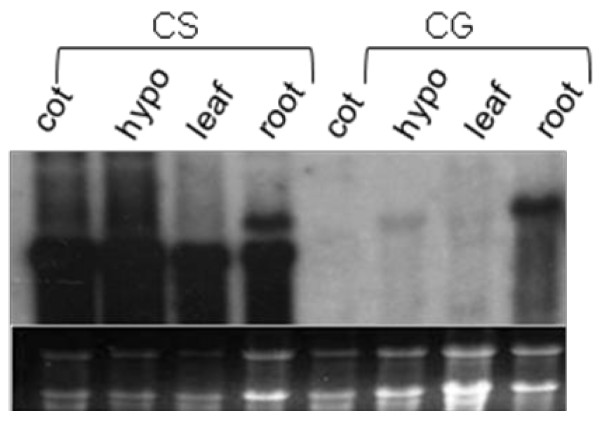
**RNA gel blot analysis of the *Glyma04g35130 *BURP gene in different organs of Clark standard and Clark glabrous**. Ten microgram of total RNA was electrophoressed through 1.2% agarose/1.1%formaldehyde gel, blotted to nitrocellulose. The cDNA probe corresponding to the *Glyma04g35130 *was labeled and hybridized.

To validate the transcriptome data for the BURP gene, we performed RNA blot analysis for the *Glyma04g35130 BURP *gene. Total RNA was isolated from mature soybean tissues and the probe was amplified from *Glyma04g35130 BURP *EST: Gm-r1083-3435. RNA blots performed on cotyledon, hypocotyl, leaf, and root organs revealed that the *Glyma04g35130 BURP *gene had strong transcript level differences among different organs in CS and CG, which validated the DGE data (Figure 4). The presence of two bands in CS root tissue might be explained by cross hybridization of the probe to more than one of the seven *BURP *genes present in the soybean genome as the BURP EST showed seven matches when used as a blast against the soybean reference genome [34] using TBLASTN program. The seven Glyma models that correspond to each feature were identified: *Glyma04g35130*, *Glyma04g08410*, *Glyma06g01570*, *Glyma06g08540*, *Glyma07g28940*, *Glyma14g20440*, and *Glyma14g20450*.

### DNA blot comparison of the *Glyma04g35130 BURP *gene in Clark standard and Clark glabrous

**Figure 5:**
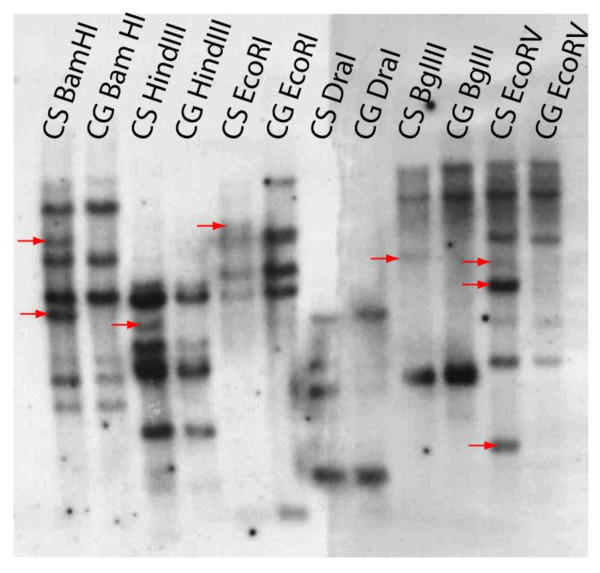
**DNA blot of Clark standard (CS) and Clark glabrous (CG) genomic DNA**. The CS and CG genomic DNA were digested with *BamHI, HindIII, EcoRI, DraI, BglII*, and *EcoRV*. The RFLPs between CS and CG digests are indicated with red arrows. The probe was a labeled cDNA corresponding to *Glyma04g35130*.

DNA blot analysis was carried out to identify potential BURP gene RFLPs between CS and CG isolines. The same cDNA PCR product used as a probe in RNA blots was used for the *Glyma04g35130 BURP *gene DNA blots. Genomic DNA was digested with six different restriction enzymes (*BamH*I, *Hind*III, *EcoR*I, *Dra*I, *Bgl*II, and *EcoR*V) and taken through the DNA blot protocol. The resulting blot shows several bands in the CS digests, not seen in the CG samples (Figure 5). These apparently missing bands may represent an insertion/deletion (indel) in the *Glyma04g35130 BURP *gene or in BURP gene family members, which is elucidated further by direct sequence analysis (below).

### Sequence Analysis of *Glyma04g35130 *BURP Gene of Clark standard and Clark glabrous

**Figure 6:**
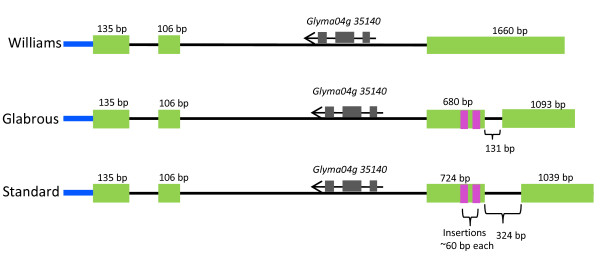
**Diagram of *Glyma04g35130 BURP *genes from cv. Williams 82, Clark standard (CS), and Clark glabrous (CG) showing structural differences**. *Green boxes *represent exons and *pink boxes *indicate insertions in the third exon. *Blue and black lines *indicate 5'UTR and introns.

**Figure 7:**
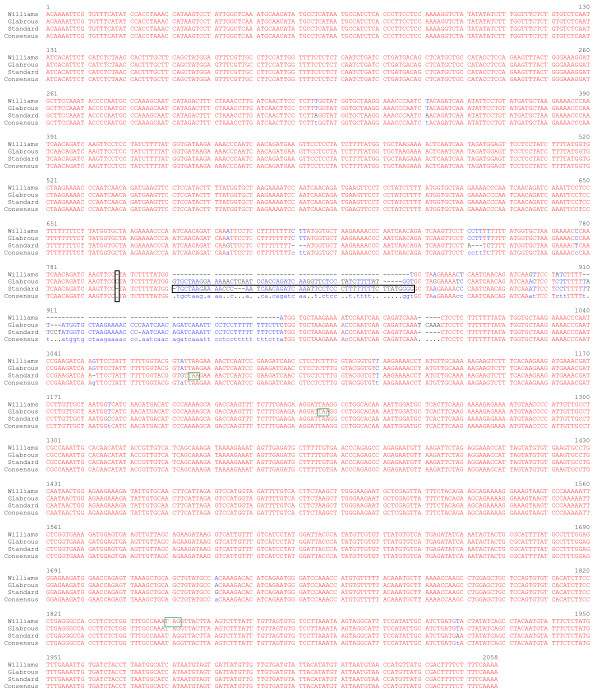
**Alignment of the *Glyma04g35130 *BURP transcript sequences from cv. Williams 82 with Clark standard (CS) and Clark glabrous (CG)**. Identical nucleotides are shown in *red*. *Dashes *represent gaps introduced for alignment. *Black boxes *represent insertions (that disrupt the reading frame) resulted in premature stop codons in CS and CG compared to Williams 82. Stop codons are indicated in *green boxes*.

**Figure 8:**
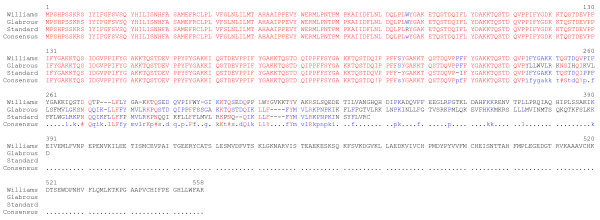
**Alignment of the deduced *Glyma04*g35130 BURP amino acid sequence from cv. Williams 82, Clark standard (CS) and Clark glabrous (CG)**. Identical amino acids are shown in *red*. The Williams 82 Glyma04g35130 peptide is 558 amino acids long where as CS and CG amino acid sequences end prematurely at 329 and 386, respectively.

The *Glyma04g35130 BURP *gene sequence from cv. Williams 82 was used to design PCR primers to amplify the corresponding genomic regions in both CS and CG. To determine the gene structures in CS and CG, the cDNA sequence was produced from RT-PCR using primers within the 5' and 3' untranslated regions for *Glyma04g35130*. Sequencing of these fragments indicated that the *Glyma04g35130 BURP *gene in CS and CG contains an additional exon and intron, for a total of four exons and three introns (Figure 6), relative to the cv. Williams 82 sequence. The comparison of cv. Williams 82 *Glyma04g35130 BURP *transcript sequence with those of CS and CG revealed various single-nucleotide polymorphisms (SNPs) and indels including two insertions of around 60 bp at positions 811 and 911 in the third exon of both CS and CG. From these two insertions, the first insertion created a premature stop codon in the transcript and resulted in a frameshift in the peptide sequence of CS; addition of one nucleotide C at position 798 in CG causes a frameshift mutation that results in premature stop codon in CG transcripts (Figure 7) and peptides (Figure 8). Extensive sequence analysis revealed that two insertions in CS and CG are actually repeats, a prominent feature of BURP domain containing genes (Figure 7). Surprisingly, the last intron of the *Glyma04g35130 BURP *gene in cv. Williams 82, CS, and CG contains another predicted gene-*Glyma04g35140*, encoding spermidine synthase (Figure 6).

**Figure 9:**
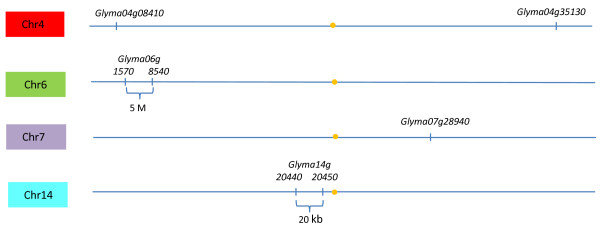
**The potential BURP gene family members with similarity to the *Glyma04g35130 *BURP EST shown as Glyma models in Phytozome and their chromosome locations**.

However, the sequence differences between the CS and CG *Glyma04g35130 *gene do not account for all the potential RFLPs seen in the DNA blots. Likely this is explained as the EST probe used for RFLP showed several matches in the soybean reference genome [34] when used as a blast that could reflect unaccounted RFLPs in the DNA blots (Figure 5). Seven potential BURP gene family members were found in the reference soybean genome [34] and these BURP gene family members are scattered on various chromosomes in the soybean genome (Table 2 & Figure 9) as expected since soybean is a an ancient tetraploid. The gene models that showed varying degrees of similarity with the probe were analyzed in DGE and RNA-Seq data to check their differential gene expression (Table 2). Among them we again found the *Glyma04g35130 BURP *gene located on the chromosome 4, with high identity to the BURP probe and also expressed differentially in CS and CG (CS/CG = 2,545/1.06 tags). The remaining seven BURP domain containing genes that showed significant similarity with the lowest e values to the BURP EST probe in phytozome do not show expression differences between CS and CG (Table 2).

### Expression analysis of soybean orthologs to known genes involved in trichome development reveal low transcript levels in young shoot tips of both lines

The genes involved in the initiation of trichome development have been particularly well characterized in Arabidopsis. The *GL1-TTG1-GL3/EGL3 *transcription factor complex has been posited to play a role in trichome development as mutations in these genes result in loss of trichomes [43-45]. We sought to look at differential expression of genes that are positive and negative regulators of trichome development in both lines (Table 3). Expression of these orthologs is very low as determined by RNA-Seq and DGE data. None of the genes described from previous reports as essential for trichome development showed higher transcript counts in our DGE data and RNA-Seq data, and likewise did not vary substantially. For instance, in the DGE transcriptome from shoot tip, the expression of *GL1 GL2*, *GL3*, and *TTG1 *showed the opposite trend with some exceptions (Table 3). One explanation to this discrepancy is that trichome development commences at a very early stage of leaf development, even before the leaf primordial is differentiated, so that these transcription factors might have been differentially expressed at higher levels at earlier stages of development of the trichomes. Thus, our DGE and RNA-Seq data may reflect genes that are expressed preferentially in trichomes and not necessarily in the early signaling stages of trichome formation.

**Table 3:** Comparison of DGE and RNA-Seq expression in soybean Clark standard and Clark glabrous of genes influencing trichome development in Arabidopsis

		DGE			RNASeq		
			
Trichome genes	Soybean orthologs	CS	CG	CS/CG	CS	CG	CS/CG
GL1	Glyma07g05960	2.8	4.5	0.6	0.5	3.8	0.1
GL3	Glyma08g01810	0.9	2.1	0.4	0.7	0.7	1.0
	Glyma05g37770	0.4	1.3	0.3	1.0	0.8	1.3
	Glyma07g07740	6.8	19	0.4	0.1	0.1	1.3
	Glyma15g01960	7.2	9.1	0.8	1.7	4.7	0.4
TTG1	Glyma06g14180	29	43	0.7	5.0	10.0	0.5
	Glyma04g40610	8.5	16	0.5	2.7	5.2	0.5
	Glyma16g04930	14	13	1.1	9.9	13.7	0.7
	Glyma19g28250	15	14	1.1	15.0	15.8	1.0
GL2	Glyma07g02220	7.2	9.1	0.8	2.6	5.9	0.4
	Glyma07g08340	21	17	1.3	4.8	6.9	0.7
	Glyma15g01960	7.2	9.1	0.8	1.7	4.7	0.4
	Glyma08g21890	14	21	0.7	2.7	4.8	0.6
	Glyma08g06190	11	4	2.7	0.1	0.2	0.5
SPL9	Glyma03g29900	37	56	0.7	4.8	4.7	1.0
	Glyma19g32800	0	0	0	8.7	9.0	1.0
TRY	Glyma06g45940	8.7	21	0.4	0.4	3.9	0.1
TCL1	Glyma11g02060	0	0.6	0	0	0	0

DGE is normalized per million tags and RNA-Seq is shown in RPKM

Other studies have shown that MYB transcription factor genes *CAPRICE (CPC)*, *TRICHOMELESS (TCL1) *and *TRIPTYCHON (TRY) *are negative regulators of trichome development [46-48]. Elevated levels of *SPLs *(*SQUAMOSA PROMOTER BINDING PROTEIN LIKE*) produced fewer trichomes in Arabidopsis. *SPL9 *directly activates the expression of MYB transcription factor genes such as *TRICHOMELESS1 (TCL1) *and *TRIPTYCHON (TRY)*, which are the negative regulators of trichome development [49]. Again, no substantial differences were found between the two soybean genotypes (Table 3).

## Discussion

While microarrays have been used extensively to reveal physiological trends from transcriptome analyses of soybean developmental stages or organ systems, fewer reports to date have focused on transcriptome analysis of near isogenic lines using either microarrays [50,51] or high throughput sequence analysis [52,53]. Here we compared high throughput sequencing using Digital Gene Expression and RNA-Seq transcriptome profiles of wild-type soybean (CS) and a glabrous-mutant (CG) with the dominant *P1 *mutation in soybean. DGE produces 16-nucleotide long tags generally specific to 3' end of each mRNA that provide information on quantitative expression of genes, rare transcripts, and also reveals novel or unannotated genes. However, since DGE data often represent the 3' end, it is essential that the databases or reference genome contain that information. We found that many of the annotated gene models in the soybean gene do not extend sufficiently to represent the DGE tags and extending the models to 250 bases at the 5' and 3' ends enables many more tags to align to the models.

Compared to DGE, RNA-Seq produces even greater numbers of reads, up to hundreds of millions in one sequencing lane. The reads are also longer, generally 75 bp and correspond to the entire coding region thus giving more depth and range of coverage. The majority of the genes that are over-expressed in CS as compared to CG were also over expressed in RNA-Seq data or a vice versa but their expression fold changes were different. The use of different technology in DGE and RNA-Seq that produced 16 bp tags from 3'ends and 75 bp tags from whole transcripts, respectively, resulted in differences between DGE and RNA-Seq data. RNA-Seq is potentially a more comprehensive way to measure transcriptome abundance, composition, and splice variants, and it also enables discover of new exons or genes. Soybean has a large and highly duplicated genome, rich in paralogs and gene families. This presents a challenge when mapping DGE tags to a specific gene, since they could equally well map to the other gene homologs in the genome. Yet, both DGE and RNA-Seq data has enabled nearly the same trend of differential expression for many of the gene models.

DGE and RNA-Seq analyses of CS and CG soybean isolines revealed several hundred genes with differential expression. Among them, the *Glyma04g35130 BURP *gene had a strong transcript level differences between the two lines. Additional validation came from RNA blots, which confirmed that the *Glyma04g35130 BURP *gene was strongly expressed in CS tissues and not in the glabrous CG isolines. There are also structural (SNP) differences between the CS and CG isolines for this gene. However, the parallel of high transcript levels for trichome-containing plants breaks down for the cv. Williams 82 which has trichomes but also has a very low level of transcripts in shoot tips of the *Glyma04g35130 BURP *gene as shown by Northern blotting (data not shown). The most distinguishing structural feature difference between the *Glyma04g35130 BURP *genes in the three cultivars is the presence of the 60 bp repeats, and an additional exon in the CS and CG lines compared to cv. Williams 82, and the addition of one nucleotide C in CG as compared to the other two.

The *Glyma04g35130 BURP *gene showed high homology to the cotton gene *RESISTANCE TO DROUGHT RD22-like 1 *(*GhRDL1*) that is involved in cotton fiber initiation and is also a member of the BURP protein family. The *Glyma04g35130 BURP *gene and *SCB1*, seed coat burp domain protein 1 (*Glyma07g28940*) fall into one BURP protein family- BURPV, when 41 BURP proteins from different species were classified into 5 subfamilies [26]. *SCB1 *may play a role in the differentiation of the seed coat parenchyma cells and is localized on the cell wall of soybean [40]. But it should be noted that despite high sequence homology among the *BURP *domain containing genes, the function of each BURP protein seems to greatly vary among plants. The *Glyma04g35130 *BURP gene does not seem to have a direct role in trichome formation but the possibility is open that it may be indirectly involved in some soybean genotypes.

Although sequence comparison of transcripts from cv. Williams 82, CS, and CG showed 98% identity, but it also revealed various SNP's, insertions, and deletions in CS and CG when compared to cv. Williams 82 (Figure 7). These differences in the transcript sequences such as ~60 bp insertion in the third exon of CS and addition of one nucleotide C in CG resulted in premature stop codons and also disturbed the frame in both CS and CG (Figure 7 &8). One might also expect differences in the upstream promoter regions of the *Glyma04g35130 *BURP between CS and CG genes based on the dramatic transcript level differences between the two genotypes as shown by DGE and confirmed by RNA blotting. The number of RFLPs seen in the CS vs. CG DNA blots suggested more family members that may differ by various indels. By comparing the BURP EST probe against the cv. Williams 82 soybean genome sequence [34], seven potential *BURP *gene family members were found that have sequence homology to the probe (Table 2) but only *Glyma04g35130 *stood out as highly differentially expressed between the two genotypes. Up to 23 total genes with BURP protein domains exist in soybean [26] but only seven are related to the *Glyma04g35130 *as assessed by e value of <10^-6^.

Some genes involved in the initiation of trichome development have been particularly well characterized in Arabidopsis. As shown in Table 3, the transcript levels of soybean orthologs to some of the Arabidopsis genes were very low and did not vary considerably between the two genotypes even in the RNA-Seq data that yielded nearly 70 million mapped reads from the young shoot tips of each genotype. It may be necessary to assay earlier stages of trichome development using laser capture microdissection to find transcripts in early trichome formation in specific cell types. Alternatively, soybean may have different and undiscovered mechanisms for trichome formation.

## Conclusion

Digital gene profiling and high throughput RNA-Seq revealed thousands of genes expressed in young trifoliate shoot tips of soybean. The data show a direct comparison of both methods. Many genes show agreement of the same trend of gene expression between the isolines but the two techniques produce differences in the ratios. Both methods allowed distinguishing gene family members in many cases. Comparison of isolines delineated changes in transcript abundance between wild-type soybean and glabrous-mutant on a genome-wide scale. Many genes showed similar expression levels between the two isolines as expected but the data also delineated the genes that are over-expressed or under-expressed in CS and may provide an insight into trichome gene expression in soybean, as the CG mutants lack any non glandular trichomes. The identification of a highly expressed member of the *BURP *gene family, *Glyma04g35130*, in CS that has almost no transcript presence in CG, may indicate its involvement in trichome formation or function in certain genotypes although it is not a candidate for the dominant *P1 *locus. Orthologs for Arabidopsis genes involved in trichome development were only very weakly expressed and did not vary considerabley between the two genotypes. This study represents a first step in expanding the study of trichome genetics into the economically important soybean plant.

## Methods

### Plant Materials and Genetic Nomenclature

The two isolines of *Glycine max *used for this study-Clark standard (L58-231) (CS) and Clark glabrous (L62-1385) (CG) were obtained from the USDA Soybean Germplasm Collections (Department of Crop Sciences, USDA/ARS University of Illinois, Urbana IL). CG mutant was generated by introgression of the *P1 *glabrous mutant line (T145) into CS for six generations. Plants were grown in the greenhouse for one month and tissues were harvested and sampled from each plant including leaves (four stages from young to older leaves), shoot tips, root, hypocotyl, cotyledons, seed coats, and stem tissue. Multiple plant and tissue samples were used for each extraction in a 12 ml extraction volume. All tissues were quick frozen in liquid nitrogen and stored at -80°C. The tissues were then lyophilized and stored at -20°C.

### DGE Library Construction and Data Analysis

Shoot tips from green house grown soybean isolines: CS and CG were collected approximately 4 weeks after planting and immediately frozen in liquid nitrogen. The RNA from multiple shoot tips and leaves was extracted using a modification of the McCarty method [54] using a 12 ml protocol with phenol chloroform extraction and lithium chloride precipitation.

Library construction was carried out at Illumina, Inc., San Diego, using illumina's DGE tag profiling technology. Briefly, double-stranded cDNA's were synthesized using oligo(dT) beads and cDNA's were digested with *Nla*III or *Dpn*II restriction enzymes and ligated to defined gene expression adapter (GEX *Nla*III Adapter 1, containing another restriction enzyme *Mme*I). Following *Mme*I digestion of cDNA's, which cuts 17 bp downstream, the GEX Adapter 2 was ligated at the site of *Mme*I cleavage. The GEX Adapter 2 contains sequences complementary to the oligos attached to the flow cell surface. Tags flanked by both adapters were enriched by PCR using primers that anneal to the ends of the adapters. The PCR products were gel purified before loading onto the illumina cluster station for sequencing.

After adapter trimming, the tags were 16-nucleotide long corresponding to 3'end of the transcript. Approximately 5.2 million DGE tags were sequenced from each library and the total counts for each unique read were determined and a unique DGE ID number was assigned to each unique tag, resulting in approximately 85,000 tags for each library where at least one library contained at least 5 counts per tag. The sequences of the DGE sequence tags and counts in each library are shown in Additional File 1.

DGE tags were aligned to the 78,774 cDNA gene models (known as Glyma models) predicted from the soybean reference genome of cv. Williams 82 [33] and available at the Phtozome web site [34] using Bowtie [35]. Using a stringent criterion of 0 mismatches within the 16 nucleotide tag alignments, most of the tags aligned to the models but large numbers of tags did not. In order to retrieve alignments where the models did not call sufficient 3'UTR sequence, we extended the Glyma models at both the 5' and 3' ends by 250 bases in each direction. Of the 5.2 million raw DGE reads for each library, approximately 4.7 million aligned to the extended Glyma models. DGE data was normalized per million aligned reads.

In addition to alignments to the Glyma models, candidate soybean ESTs corresponding to the tags were used for further verification of the DGE differentially expressed tags referenced in the Table 1. First, each read was compared to the publically available soybean EST sequences available at NCBI via a BLASTN search. Each read was used to identify 100% matches, and only clones matching at least three separate ESTs were used for further analysis. The identified ESTs corresponding to each read were then compared with the non-redundant sequence database at NCBI, using BLASTX. Reads were included in the final list only if all three (or two, 100% identical to reads) had matching annotations. For differential gene expression analysis with count data using a negative binomial distribution without replication, the DESeq package in R was used [41].

### RNA-Seq Method

The RNA from multiple shoot tips was extracted using a modification of the McCarty method [45] using a 12 ml protocol with phenol chloroform extraction and lithium chloride precipitation. The shoot tips were harvested from a second biological replication of ~4-week old plants grown in green house. Library construction and high-throughput sequencing was carried out using RNA-Seq technology at using Illumina GaII instruments by the Keck Center, University of Illinois.

### RNA-Seq Allignment and Data Normalization

The 75 bp reads were mapped to the 78,744 Glyma cDNA gene models [34] using Bowtie [35] with up to 3 mismatches allowed and up to 25 alignments. A total of the 91.4 and 88.7 million reads were generated in each lane of Illumina sequencing for the CS and CG libraries, respectively. Of these, 65.4 (71%) and 70.3 (79%) million reads aligned to the 78,744 target Glyma models with the Bowtie criteria used. RNA-Seq data was normalized in reads per kilobase of gene model per million mapped reads (RRKM) as the RNA-Seq depends on the transcript length [42] as the reads will map to all positions of the transcript, unlike DGE tags which are predominantly found adjacent to the first *Dpn*II site at the 3' end of the transcript. The RNA-Seq data discussed in this publication have been deposited in NCBI's Gene Expression Omnibus [55] and are accessible through GEO Series accession number GSE33155.

### Annotation of Glyma models

Coding region gene models were collected from the masked soybean genome from Phytozome version 4.0 GFF file [34]. In addition to the PFAM, KOG and Panther annotations downloaded from Phytozome, the 78,744 models (that include both high and low confidence models) were further annotated using BLASTX against the non-redundant (nr) database of the National Center for Biotechnology Information [55] and trEMBL and Swiss prot of the European Bioinformatics Institute [56] on a Time Logic CodeQuest DeCypher Engine.

### BURP Gene Cloning and Sequence Analysis

Primers from the cv. Williams 82 genomic sequence [33,34] were used to amplify the full-length BURP gene from CS and CG genomic DNA using the primers 5' ACATCATTCTAAAAGACATAGACTA3' and 5' TGACCTGTTAGCTTTATGAT3'. A cDNA sequence was amplified from CS root tissue using RT-PCR with primers designed on 5' and 3' untranslated regions (5' CCACCTAAACCATAAGTCCTATTGG3' and 5' CCTATTACTAAAATGTAGGTTCAGTAAAGGTAG3'). All genomic and cDNA sequences were cloned and confirmed by DNA sequencing. The cDNA and genomic sequences of *Glyma04g35130 *from both lines, CS and CG were compared to determine the number of introns and exons in the gene.

### RNA Blot

Total RNA was extracted from the frozen leaves, roots, hypocotyls, seed coats, and cotyledons of CS and CG using standard phenol chloroform method with lithium chloride precipitation [54]. RNA samples were quantified by spectrophotometer and the integrity was confirmed using agarose gel electrophoresis. RNA was stored at -80°C until further use.

For RNA gel blot analysis, 10 μg of total RNA was electrophoresed through 1.2% agarose/1.1% formaldehyde gels [57] blotted onto nitrocellulose membranes (Schleicher & Schuell, Keene, NH) via capillary action with 10× SSC (1.5 M NaCl and 0.15 M sodium citrate, pH = 7) overnight. After blotting, RNA was cross-linked to the nitrocellulose membranes with UV radiation by a UV cross-linker (Stratagene, La Jolla, CA). Nitrocellulose RNA gel blots were then prehybridized, hybridized, washed, and exposed to Hyperfilm (Amersham, Piscataway, NJ) as described by Todd and Vodkin (1996) [58].

A 1.4 kb probe for BURP gene was amplified from EST (Gm-r1083-3435) and labeled with [α-^32^P]dATP by random primer reaction method [59].

### DNA Blot

For DNA blots, genomic DNA was isolated from lyophilized soybean shoot tips using the method described by Dellaporta in 1993 [60] with minor modifications. Genomic DNAs were digested with six different restriction enzymes including *BamH*I, *Hind*III, *EcoR*I, *Dra*I, *Bgl*II, and *EcoR*V in separate reactions. Ten micrograms of digested genomic DNA from each sample was separated on 0.7% agarose gels. The gels were then treated sequentially with depurination solution (0.25 M HCl), denaturation solution (1.5 M NaCl, 0.5 M NaOH), and neutralization solution (1 M Tris, 1.5 M NaCl [pH 7.4]). The gels were then taken through the same blotting transfer protocol described above for Northern blots along with prehybridization, hybridization (with the appropriate [α-^32^P]dATP labeled probed), washing, and exposure to Hyperfilm (Amersham, Piscataway, NJ). The same EST probe used for RNA blot was used in the DNA blots.

## Authors' contributions

MH designed experiments, performed RNA and DNA extractions and blots, amplified and sequenced BURP gene from CS and CG genotypes, analyzed DGE data for functional categories, and drafted the manuscript; NK performed transcript cloning, RNA blots, analyzed DGE data using DESeq software, analyzed RNA-Seq data, BURP genome sequence data, and drafted sections of the manuscript; MS annotated Glyma models with multiple databases. LOV designed initial approach, led and coordinated the project, and edited the manuscript. All authors have read and approved the final manuscript.

## Supplementary Material

Additional file 1 **Alignment of DGE tags to extended Glyma model and their annotations**. Click here for file

Additional file 2 **The top 300 genes that are highly expressed in Clark standard and Clark glabrous**. Click here for file

Additional file 3 **Differential expression from DGE and RNA-Seq of Clark standard and Clark glabrous**. Click here for file

Additional file 4 **DESeq analysis of Clark standard and Clark glabrous**. Click here for file
